# Immunomodulatory Effects of Halichondrin Isolated from Marine Sponges and Its Synthetic Analogs in Oncological Applications

**DOI:** 10.3390/md22090426

**Published:** 2024-09-20

**Authors:** Dinusha Shiromala Dissanayake, Dineth Pramuditha Nagahawatta, Jung-Suck Lee, You-Jin Jeon

**Affiliations:** 1 Department of Marine Life Sciences, School of Marine Biomedical Sciences, Jeju National University, Jeju 63243, Republic of Korea; dinudissanayake95@stu.jejunu.ac.kr (D.S.D.); pramuditha1992@jejunu.ac.kr (D.P.N.); 2 Department of Seafood Science and Technology, Institute of Marine Industry, Gyeongsang National University, Tongyeong 53064, Republic of Korea

**Keywords:** halichondrin, eribulin, cancer therapeutics, E7389, microtubule disruption

## Abstract

Marine natural products comprise unique chemical structures and vast varieties of biological activities. This review aims to summarize halichondrin, a marine natural product, and its synthetic analogs along with its therapeutic properties and mechanisms. Halichondrin and its analogs, derived from marine sponges, exhibit potent antineoplastic properties, making them promising candidates for cancer therapeutics. These compounds, characterized by their complex molecular structures, have demonstrated significant efficacy in inhibiting microtubule dynamics, leading to cell cycle arrest and apoptosis in various cancer cell lines. Several types of halichondrins such as halichondrins B, C, norhalichondrin B, and homohalichondrin B have been discovered with similar anticancer and antitumor characteristics. Since naturally available halichondrins show hurdles in synthesis, recent advancements in synthetic methodologies have enabled the development of several halichondrin analogs, such as E7389 (eribulin), which have shown improved therapeutic indices. Eribulin has shown excellent immunomodulatory properties by several mechanisms such as reprogramming tumor microenvironments, facilitating the infiltration and activation of immune cells, and inhibiting microtubule dynamics. Despite promising results, challenges remain in the synthesis and clinical application of these compounds. This review explores the mechanisms underlying the immunomodulatory activity of halichondrin and its analogs in cancer therapy, along with their clinical applications and potential for future drug development.

## 1. Introduction

The ocean, which comprises 70% of the Earth’s surface, is an enormous reservoir of discovered and undiscovered biological molecules with numerous bioactivities. Marine natural products refer to a broad range of bioactive substances from marine life, including bacteria, fungi, algae, sponges, and corals [1]. These special compounds, with their remarkable chemical variety and powerful biological activity that frequently outweigh their terrestrial counterparts, have been the focus of scientific inquiry. With its wide range of ecosystems, the marine environment is home to an undiscovered supply of cutting-edge chemicals that could be used in biotechnology, pharmaceuticals, and other industries.

This review investigates halichondrins, which were first identified and isolated from a species of Japanese marine sponge, *Halichondria okadai* Kadota, in 1986 [2], their derivatives, and their promising use in oncology. The mode of action of halichondrin and its synthetic analogs uniquely disrupts the microtubule dynamics compared to other microtubule-targeting agents. This leads cancer cells to cell cycle arrest and apoptosis. These have shown excellent activity against many types of cancer [3]. Therefore, these intricate polyether macrolides have attracted much attention because of their exceptional antitumor effects. The most well-known halichondrin, halichondrin B, disrupts the dynamics of microtubules, a process essential to cell division, exhibiting strong cytotoxic action. Anticancer drugs taxanes and vinca alkaloids share a similar mechanism of action. Halichondrins have a distinct structure and way of binding that opens new therapeutic options for cancer treatment [4]. However, due to their complex and large molecular structure, halichondrin manufacture and research have been extremely difficult. Despite these difficulties, developments in synthetic chemistry have produced analogs such as eribulin mesylate, licensed for treating liposarcoma and metastatic breast cancer [5]. This approval highlights the halichondrin derivatives’ potential as useful medication in oncology, providing hope for better treatment choices against a range of cancers.

The control of immune system responses, or immunomodulation, is essential for treating many kinds of illnesses, such as cancer, infections, autoimmune diseases, transplant rejection, and persistent inflammatory diseases [6]. Immunomodulatory treatments try to bring the immune system back into balance and enhance or decrease immunological function. Immunomodulation is beneficial in recognizing and attacking cancer cells efficiently since it manipulates the immune system accordingly in cancer therapy. Unlike traditional cancer treatments such as chemotherapy and radiation, immunomodulatory approaches enhance the body’s natural defenses to attack cancer by improving outcomes and potential side effects. Immune checkpoint inhibitors enhance antitumor immunity in cancer, while monoclonal antibodies diminish inappropriate immune responses in autoimmune disorders [7].

The current review comprehensively discusses the bioactivity of halichondrins and their derivatives in oncological applications by filling several gaps in the current literature by outlining the primary sources of information and combining the obtained data. In addition, this study reviews newer insights into the synthesis of these compounds and their clinical uses, focusing on the issues and prospects.

## 2. Marine Sponges as a Source of Halichondrins

Being the simplest multicellular organism, marine sponges possess lightly organized single cells without distinguished true tissues or organs. They often have numerous bioactive compounds that can fight inflammation and cancer without showing side effects [8]. Among different genera, 11 genera of sponges have shown bioactive substances with strong anticancer properties [9]. Halichondrins are frequently discovered in marine sponges, especially those belonging to the genus *Halichondria* [10]. The animal phylum *Porifera*, class *Demospongiae*, subclass *Heteroscleromorpha*, order *Suberitida*, and family *Halichondriae* are home to the genus *Halichondria*. Encrusting, huge, occasionally irregularly branched, or digitated sponges with smooth or papillate surfaces are examples of *Halichondria* species’ growth types [11].

The isolation of halichondrins involves several steps such as crushing the samples with an organic solvent such as methanol and generating the crude extract from the source by subjecting it to organic solvent extractions like n-butanol-based extractions, followed by multiple rounds of fractionations using various chromatographic techniques, such as reverse-phase high-performance liquid chromatography (HPLC), silica gel chromatography, and gel filtration, to obtain a single compound [2]. Spectroscopic methods and chemical analysis were combined to determine the structure of halichondrins. The structure was ascertained using nuclear magnetic resonance (NMR) spectroscopy, which encompasses both 1D and 2D methods (COSY, HSQC, and HMBC). This made it possible for scientists to pinpoint the locations of the carbon and hydrogen atoms within the molecule and to clarify how various functional groups are related to one another [12]. The molecular formula and the existence of subunits were confirmed with the aid of mass spectrometry, which also provided information on the molecular weight and fragmentation pattern of halichondrins. X-ray crystallography has occasionally been used to ascertain the three-dimensional structure of halichondrins [13]. This method gave a clear image of how the atoms are arranged in space within the molecule. The locations of specific functional groups were inferred, and the suggested structure derived from NMR and MS data was confirmed through chemical modification and degradation studies.

### 2.1. Structural Features of Halichondrins

A macrolactone spanning C1–C30, two exo-methylene groups at C19 and C26, spiroketals at C38 and C44, and a unique tricyclic 2,6,9-trioxatricyclo [3.2.2.0^3,7^] decane “caged” ketal found between C8 and C14, which includes the C-, D-, and E-rings, are all present in halichondrin B’s incredibly diverse structure [14]. Halichondrins’ unique multiple ether bonds which are oxygen atoms linking to two carbon atoms (C–O–C) form large and complex ring structures defining their characteristic 3D shape and forming macrocyclic rings [10]. These macrocyclic rings are a distinct feature of polyether, and they contribute to the rigidity and conformational stability of the molecule. Many fused and spirocyclic rings can be found in halichondrins and these rings are joined by a single carbon atom, whereas fused rings share two nearby carbon atoms. These intricate ring structures enhance the molecule’s three-dimensional form and affect how it interacts with biological targets [10]. The polyether rings of halichondrins include numerous chiral centers, which are carbon atoms attached to four different groups. The specific spatial arrangement of these chiral centers (stereochemistry) is crucial for the biological activity of halichondrins, as it affects how the molecule fits into the binding sites of its biological targets. Numerous functional groups, including hydroxyl (-OH), methyl (CH_3_), and methoxy (OCH_3_) groups, are joined to the polyether rings [11]. The activity and solubility of the molecule can be further influenced by these groups’ participation in hydrophobic interactions, hydrogen bonding, and other chemical interactions. Because the ethers are cyclic, halichondrins have a polyether structure with both hard segments and flexible linkers that permit some conformational flexibility. The flexibility and stiffness balance are crucial for the molecule’s capacity to interact with many biological targets [12]. 

### 2.2. Notable Types of Halichondrins

Despite sharing a structural similarity as complex polyether macrolides, different halichondrin types differ in ways that greatly affect their biological activity and their therapeutic uses. The following are some noteworthy halichondrin subtypes that have been isolated from marine sponges and are identified by their distinct structural variants and biological activities.

#### 2.2.1. Halichondrin B

**Figure 1:**
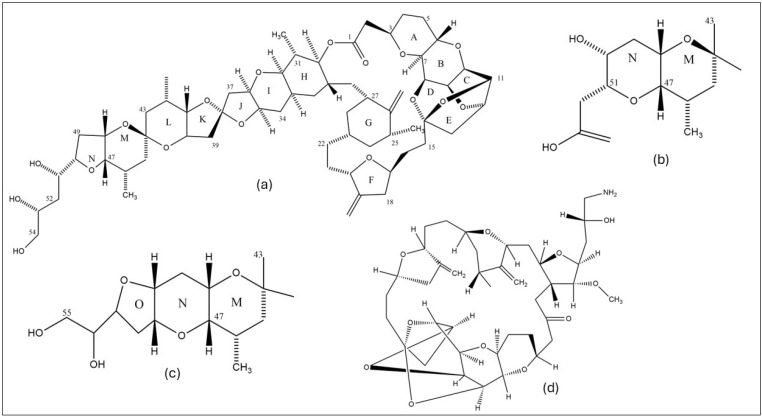
Chemical structures of different types of halichondrin and its synthetic analog, (**a**) halichondrin b, (**b**) norhalichondrin b, (**c**) homohalichondrin b, (**d**) eribulin.

Due to the presence of extraordinary cytotoxicity against cancer cells, the discovery of halichondrin B (Figure 1a) was a significant milestone in the field of marine pharmacology. The main way that halichondrin B works is by preventing the construction of microtubules, which is essential for mitosis and other cellular processes [15]. The natural abundance of halichondrin B is incredibly low, despite its potency, which makes it difficult to collect significant quantities for in-depth research and therapeutic application from natural sources. Chemists pursued the complete synthesis of halichondrin B and its analogs as a result of this scarcity. Notably, eribulin mesylate, also known as Halaven, is a synthetic derivative that is easier to make while maintaining the biological action and essential structure of halichondrin B [16].

#### 2.2.2. Halichondrin C

Closely linked to halichondrin B, halichondrin C is a physiologically active and structurally complex marine natural product. Similar to halichondrin B, halichondrin C demonstrates strong anticancer effects, mainly by disrupting the dynamics of microtubules. Because this interference prevents microtubules from properly forming and functioning, it limits cell division and causes cancer cells to undergo cell cycle arrest and apoptosis [12]. The great specificity and effectiveness of halichondrin C as an anticancer agent are attributed to its complex chemical structure, which is characterized by many rings and oxygen atoms [13]. However, large-scale isolation and synthesis of halichondrin C also present difficulties because of its low natural abundance and intricate structure. These challenges highlight the necessity for sophisticated synthetic techniques and motivate continued research into synthetic analogs and derivatives with the goal of better using its medicinal potential.

#### 2.2.3. Norhalichondrin B

A lesser-known member of the halichondrin family of natural products, which is well-known for its intricate structures and strong biological effects, is norhalichondrin B (Figure 1b). This chemical is obtained from sea sponges of the genus *Halichondria*, just like its relatives. The particular sponges that produce norhalichondrin B can be found in a variety of marine habitats, such as the Pacific Ocean and the coastal seas of Japan [17]. Norhalichondrin B’s chemical structure is distinguished by its polyether macrolide framework, which differs somewhat from other halichondrins because of changes that give it a special bioactivity. The structural variation is reflected in its name, where “nor” denotes the absence of a certain group in comparison to the parent halichondrin complex [18]. The anticancer characteristics of norhalichondrin A are similar to those of the halichondrin family. Norhalichondrin B is difficult to acquire in considerable quantities due to its complicated structure and low natural abundance. This means that sophisticated synthetic procedures are needed for future research and possible medicinal usage [19]. Norhalichondrin B research, as well as research on other halichondrins, highlights the importance of marine natural products in medicine development. These substances stimulate the manufacture of analogs that may be developed into potent cancer medicines in addition to offering insightful information about the processes behind cell division and cancer growth.

#### 2.2.4. Homohalichondrin B

A prominent member of the halichondrin family, homohalichondrin B (Figure 1c) is renowned for both its strong biological activity and complex molecular structure. It is isolated from sea sponges, namely, those belonging to the *Halichondria* genus, just like other halichondrins. Homohalichondrin B is a complicated polyether macrolide with many oxygen atoms and large ring systems in its chemical structure. Comparing this structure to halichondrin B reveals some modifications that add to its distinct biological characteristics [20]. In contrast to halichondrin B, it has one extra methylene group in its structure, as indicated by the term “homo” in its name. The limited natural abundance of homohalichondrin B makes it challenging to collect significant quantities for in-depth research and clinical usage, despite its potential. This difficulty has increased interest in semi-synthetic and synthetic methods for the synthesis of analogs of homohalichondrin B [21]. These initiatives seek to overcome the drawbacks of its natural extraction while maximizing its medicinal potential. The growth and investigation of homohalichondrin B and associated substances highlights the significance of marine natural products in the process of developing new drugs. In addition to providing new perspectives on how to combat cancer, they also serve as a source of inspiration for the synthesis of synthetic derivatives that may be used to create potent cancer therapies, such as eribulin, which is derived from halichondrin B.

#### 2.2.5. Other Types of Halichondrins

Apart from the previously mentioned halichondrin types, researchers have discovered several halichondrin types that are not commonly found. A study has reported two types of halichondrins known as halistatin 1 and 2 discovered from *Phakellia carteri*, an Indian Ocean sponge. Another new halichondrin type known as isohomohalichondrin whose main difference from halichondrin B was said to exist beyond C48 was extracted from *Lissodendoryx* n. sp. [9]. Table 1 summarizes general information about commonly available halichondrin types.

### 2.3. Comparison with Synthetic Analogs

Even though the naturally occurring halichondrins show higher therapeutic potential, their inherent structural complexity and the difficulty of harvesting adequate quantities from natural sources limit their direct use in clinical applications.

A novel 2,6,9-trioxatricyclo [3.3.2.0^3,7^] decane system (rings C–E), a 22-membered lactone ring (C1–C30), two exocyclic olefinic groups, and many pyranose and furanose rings are among the distinctive structural features of the halichondrin compounds. The level of oxidation at positions C12 and C13 distinguishes the halichondrin A, B, and C families, with additional variants occurring beyond the C45 location [9]. Another technique for producing significant amounts of these chemicals is the complete synthesis of halichondrins, however, this is a difficult and demanding process. In 1992, Kishi’s group reported the first complete synthesis of norhalichondrin B and halichondrin B. This process required more than 100 reaction steps and relied on low-cost starting materials with absolute stereochemistry understood [27].

The insufficient amount of the natural substance initially hampered efforts to develop halichondrin B as an anticancer medication. Nevertheless, scientists created a synthetic, structurally simplified derivative that kept the original compound’s biologically active macrocyclic lactone C1 to C38 moiety and high potency. The pharmacologic actions of this derivative, called eribulin, are mediated by binding to the plus ends of microtubules, inhibiting microtubule growth without changing microtubule shortening, and causing nonproductive tubulin aggregates to develop [28]. Unlike other tubulin-targeting medications like taxanes, epothilones, and vinca alkaloids, this mode of action is unique.

The effective synthesis of halichondrin B by the Kishi laboratory in 1992 was a major milestone. This accomplishment made it possible to design, synthesize, and assess a number of analogs, such as eribulin (ER-086526, E7389, NSC-707389) (Figure 1d) [29]. Eventually, eribulin’s difficult 63-step synthesis was rendered commercially viable. After more than 180 halichondrin B analogs were synthesized, two macrocyclic ketone analogs with the best preclinical activity were found. The C35 primary amine-substituted molecule, eribulin, was chosen for clinical development following a thorough preclinical testing procedure [30].

#### Other Notable Analogs

There have been attempts to create additional synthetic analogs of halichondrin B besides eribulin. These analogs seek to preserve the native product’s anticancer action while streamlining its intricate structure for simpler manufacturing and possibly enhancing its pharmacological characteristics.

To improve the transport and effectiveness of this powerful anticancer medication, eribulin has been liposomally encapsulated in a unique form called E7389-LF (eribulin liposomal formulation) [31]. A synthetic version of halichondrin B, eribulin has potent anticancer properties by blocking microtubule dynamics, which is essential for cell division. Through increased stability and bioavailability, decreased systemic toxicity, and tailored distribution to tumor sites, the liposomal formulation seeks to improve the pharmacokinetic profile of eribulin. By reducing side effects and maybe lengthening the therapeutic window, this encapsulation method makes E7389-LF a promising development in the treatment of different malignancies [32]. Derived from the natural substance halichondrin B, E7130 is another new anticancer agent. To improve the potency and pharmacological qualities of this molecule, structural modifications have been made. E7130 operates by attaching to microtubules and causing them to undergo a state of disruption that is essential for cell division and proliferation [33].

### 2.4. Synthetic Strategies Used in Total Synthesis of Halichondrins

Total synthesis refers to the complete chemical synthesis of halichondrin. Confirming the structure of a natural product was a primary goal of complete synthesis in the early 20th century. The process of giving naturally existing substances a molecular structure before the development of complete synthesis was quite difficult and depended on antiquated tools and methods.

Several studies have been conducted on the synthesis of halichondrin and its analogs, and the strategy of developing the halichondrin structure varies depending on the experimental procedure. In general, the synthesis of halichondrins involves several major steps, including constructing the intricate macrocyclic core and elaborating the peripheral side chains [34]. The accurate creation of several stereocenters and the effective assembly of the macrocyclic framework are two major challenges in the synthesis of halichondrin. These goals are frequently met by convergent or linear synthetic pathways that make use of cutting-edge methods like macrolactonization, Suzuki couplings, and aldol reactions [22].

One key step is retrosynthetic analysis where the complex structure of halichondrin is broken down into simpler building blocks. Usually, the building blocks are separated into two parts, the macrocyclic region which starts from C1 to C38 and the spirocyclic region which starts from C39 to C54 [10]. Due to the complexity in the halichondrin structure, the synthetic process focuses on recognizing the main disconnections to simplify the structure using several strategies such as macrocyclization, fragmentation of the polyether regions, utilization of aldol and ether-forming reactions, and key functional group disconnection, particularly at points like C-glycosidic bonds and cyclic ethers. Another strategy used in the synthesis of halichondrins is fragment-based synthesis in which the halichondrin structure is broken down into more manageable fragments that can be synthesized independently and later strategically coupled using advanced chemical reactions [34]. Key reactions play a major role that allows for the construction of its highly intricate molecular structures. Polyketide chain assembly is one such reaction that is used to construct the polyether backbone of halichondrin. This mechanism involves several reactions such as aldol reactions, aldol-like additions, and Witting reactions to assemble C–C bonds [35]. Macrocyclization is another step that forms the large macrocyclic ring. The macrocycle is normally closed via Nozaki–Hiyama–Kishi coupling or ring-closing metathesis (RCM), which guarantees appropriate stereochemistry and relieves ring strain [35]. Halichondrins are composed of many ether linkages, which are frequently created via oxy-Michael additions or epoxide-opening processes. These processes contribute to the formation of the polyether framework that is unique to halichondrins. Halichondrins’ stereochemistry is essential to their bioactivity. Stereocenters are introduced and controlled in molecules using enantioselective reactions such as Evans aldol reactions, Brown allylation, and Sharpless asymmetric epoxidation [36]. Owing to the intricate structure, connecting smaller fragments is frequently required throughout the synthesis process. Larger subunits can be joined by Suzuki–Miyaura coupling or Julia–Kocienski olefination, particularly to form C–C bonds between advanced intermediates [37].

Numerous groups reported conducting synthetic research on halichondrin members. Out of all of them, the Kishi group was the first to create a complete synthesis of halichondrin B in 1992 [38]. The four subunits that their highly convergent technique unites are the C27–C38 fragment, the C14–C26 fragment, the C1–C13 fragment, and the C39–C54 fragment. These fragments were all made from easily accessible starting materials based on carbohydrates. The fact that each module allowed for separate alterations was significant since it opened the door for the creation of simpler analogs. This suggests that additional simplification of the eastern region might be carried out without significantly reducing the activity, as it was demonstrated that the observed growth inhibitory effect was exclusively attributed to the right-half macrolactone fragment [39].

For the complete synthesis of halichondrin B, a novel methodology is presented that involves reversing the classical method’s sequential creation of several of its cyclic ethers and instead generating C–O bonds first, followed by C–C bond formation. By utilizing the Nicholas reaction to produce linear ethers, which serve as precursors for the complete synthesis of halichondrin B and other compounds belonging to the halichondrin and eribulin families, this innovative method offers fresh prospects for the advancement of enhanced synthesis of these intricate and valuable compounds. This method involves reversing the order of certain C–O and C–C bond formation times during the synthesis process. In other words, instead of forming the C–C bonds first and then the C–O bonds to construct the necessary cyclic ether moieties, the reverse strategy uses the Nicholas reaction to first construct the C–O bond of several cyclic ether structural motifs, and then a radical-induced ring closure to complete several of the cyclic ether systems of the target molecule [38].

The most difficult halichondrin family member discovered to date, complete synthesis neonorhalichondrin B(1), was described in a different study. Blunt, Munro, and associates isolated neonorhalichondrin B as a minor ingredient from *Lissodendoryx* sp. that was collected off the coast of New Zealand, together with a number of other known and unknown halichondrins. From a structural standpoint, it stands out due to its distinct left-half oxidation pattern and the strangely bridged bicyclic ring structure that connects C44 to C49. Based on the previously reported model work, they have initiated the synthesis of neonorhalichondrin B (49R, 51S, 52S). Using their recently reported Ni(I)/Ni(II)-mediated ketone-coupling conditions for the union of left-half C38 thiopyridyl ester 6 with right-half C37 iodide, the synthesis of neonorhalichondrin B(1) depended on a late-stage nickel-catalyzed ketone coupling reported in the unified synthesis of the halichondrin family of natural products. They predicted that a second nickel-catalyzed ketone coupling with a known iodide may yield the C38 thioester, which could then be produced via acid-catalyzed spiroketalization. In turn, epoxide and iodide can be used to create precursor C44 thioester. The process has been continued with the synthesis of an epoxide-coupling partner, C44 thioester, and ketone coupling [40].

The whole synthesis of eribulin and a macrolactam analog of halichondrin B are disclosed using a single synthetic pathway. The utilization of our reverse approach for the creation of cyclic ether structural motifs and a modified intramolecular cyclization reaction between alkyl iodide and aldehyde functionalities to establish the all-carbon macrocyclic framework of eribulin are crucial components of the current synthetic approach. These syntheses validate and demonstrate the powerful reverse strategy in the building of cyclic ether structural motifs, as does our previous work on the entire syntheses of halichondrin B and norhalichondrin B. However, inspiration and potential in the realm of halichondrin and related polycyclic ethers can be found in the unified synthetic technique for the production of the related macrolactam analog [17].

## 3. Immunomodulatory Effects and Potential Therapeutic Applications of Halichondrin and Its Synthetic Analog

Halichondrin’s strong biological effects, especially its anticancer effects, have attracted a lot of attention. Recent research has started to investigate the immunomodulatory effects of halichondrin and its derivatives in addition to its well-known cytotoxic properties [41]. These substances seem to affect immune function by adjusting the activity of different immune cells, which may improve the body’s capacity to identify and combat cancerous cells. Due to halichondrin’s immunomodulatory qualities, novel cancer treatments that combine immune system stimulation and direct anticancer action may be developed, providing a more comprehensive approach to the disease’s therapy [42]. This new field of study aims to provide further insight into the potential of halichondrin to enhance oncology treatment outcomes.

### 3.1. Enhancement of Antitumor Immunity

Halichondrins have gained much attention due to their excellent anticancer activity. Eribulin, the synthetic analog of halichondrin, promotes antitumor activity in different mechanisms. It can reprogram the tumor microenvironment (TME) via various mechanisms and promotes the infiltration and activation of immune cells [43]. Another well-known function of eribulin is that it can inhibit microtubule dynamics [31].

#### 3.1.1. Reprogramming Tumor Microenvironment

Reprogramming the TME involves various mechanisms, and they collectively contribute to therapeutic efficacy. It has been demonstrated that eribulin remodels the tumor vasculature, improving oxygenation and perfusion inside the tumor. The process of restoring the aberrant structure and function of blood vessels within tumors to a more normal condition is known as “normalization of tumor vasculature” [44]. The aberrant tumor microenvironment is characterized by hypoxia (low oxygen levels), high interstitial pressure, and poor transport of nutrients and therapeutic medicines. Tumor blood vessels are generally irregular, disordered, and leaky. Normalization of these tumor blood vessels occurs in different aspects. One such aspect involves straightening and stabilizing the tumor blood vessels, reducing the number of abnormal branches, and improving the overall architecture [45]. Hypoxia is lessened because of this action, which frequently encourages tumor growth and treatment resistance. Reduced hypoxia results from normalized arteries, which improve blood flow and oxygen supply inside the tumor. This may increase the potency of chemotherapy and radiation therapy, which depend on oxygen to produce free radicals that destroy cancer cells [46].

Additional therapeutic medicines can be delivered to the tumor site more effectively with improved tumor perfusion. Eribulin can alter the tumor’s immunological environment. It has been noted to decrease the quantity of immune-suppressive cells in the TME, including myeloid-derived suppressor cells (MDSCs) and regulatory T cells (Tregs). Effector immune cells, such as cytotoxic T lymphocytes, which oversee attacking and eliminating cancer cells, may become more active because of this decrease [31].

Cancer-associated fibroblasts (CAFs) play an important role in the progression of cancer by promoting tumor development, invasion, metastasis, and resistance to medications and therapies. Eribulin can cause cancer cells to change phenotypically from a mesenchymal state linked to increased invasiveness and resistance to treatment to an epithelial state that is less invasive and more amenable to therapy. This mechanism, called MET, can make cancer cells more sensitive to other therapies and less likely to spread. Also, eribulin has been shown to have inhibitory effects on fibroblasts, preventing the fibroblasts from converting into CAFs. Activated CAFs release growth factors, cytokines, and extracellular matrix components that promote tumor growth and metastasis [47]. They also display markers such as alpha-smooth muscle actin (α-SMA). Eribulin interferes with the supporting function of CAFs in the TME by blocking this activation [48]. Eribulin’s effects on CAFs have been shown to contribute to its observed decrease in stromal density. Denser stroma can protect tumor cells by obstructing the delivery of therapeutic medicines to cancer cells. Eribulin improves drug delivery and lessens the stromal density which provides a shield for cancer cells [44].

Eribulin may lower the tumor’s cancer stem cell (CSC) population. CSCs are linked to tumor spread and recurrence, and they are frequently resistant to traditional treatments. Eribulin can target these cells, which may reduce the chance of relapse and enhance long-term results [49].

#### 3.1.2. Promoting the Infiltration and Activation of Immune Cells

By multiple routes, eribulin facilitates the infiltration and activation of immune cells, including natural killer (NK) cells and cytotoxic T lymphocytes (CTLs). Eribulin can cause a type of cell death. Damage-associated molecular patterns (DAMPs) are released by dying cancer cells during immune cell depletion (ICD), and these molecules can promote the recruitment and activation of dendritic cells (DCs). Following that, these DCs process and deliver tumor antigens to T cells, encouraging CTL activation and proliferation. Eribulin can improve the way that DCs and other antigen-presenting cells (APCs) deliver tumor antigens by triggering ICD and altering the TME. The priming and activation of CTLs, which enable them to identify and attack tumor cells, depend on this mechanism. According to certain research, eribulin may directly activate immune cells. For instance, eribulin has been shown to increase NK cells’ cytotoxic activity, enabling them to kill tumor cells straight away without the need for prior sensitization [50]. By using these pathways, eribulin increases its overall anticancer efficiency by directly cytotoxically attacking tumor cells and utilizing the body’s immune system to identify and combat cancer.

#### 3.1.3. Inhibition of Microtubule Dynamics

By attaching itself to the growth ends (plus ends) of microtubules, eribulin suppresses the dynamics of microtubules. By preventing tubulin subunits from joining the microtubules, this mechanism effectively inhibits the polymerization of the microtubules. Eribulin attaches itself to the dynamic ends of microtubules, or the plus ends, where tubulin dimers are often added during the microtubule polymerization process. Eribulin inhibits the formation of new tubulin dimers by attaching itself to their ends, which stops microtubule development. The result is a decrease in the microtubules’ total length [39]. Eribulin not only prevents polymerization but also encourages the depolymerization of preexisting microtubules. This indicates that when tubulin subunits are removed from their ends, microtubules start to contract. The creation of the mitotic spindle, which is essential for chromosomal segregation during mitosis, is hampered by eribulin’s disruption of this balance. In the G2/M phase, a disturbance of microtubule dynamics results in cell cycle arrest. Without functioning mitotic spindles, cells cannot complete mitosis and enter the cell cycle, which results in cell cycle arrest. Programmed cell death (apoptosis) is ultimately brought on by prolonged cell cycle stoppage and the incapacity to finish mitosis [51]. This is a key process by which eribulin inhibits the growth of tumors. Inducing apoptosis and successfully inhibiting cancer cell proliferation, eribulin disrupts microtubule dynamics, rendering it a valuable chemotherapeutic drug for the treatment of specific cancers, including liposarcoma and metastatic breast cancer.

Disruption of microtubule dynamics by eribulin indirectly affects the extracellular matrix (ECM) by altering the behavior of the cancer cells. Cancer cells interact with and modify the ECM to facilitate their invasion and metastasis. Eribulin inhibits the expression of proteins like matrix metalloproteinases (MMPs) that break down extracellular matrix (ECM). Because MMPs degrade ECM, cancer cells can infiltrate neighboring tissues. Eribulin aids in stabilizing extracellular matrix (ECM) by lowering MMP levels, which hinders the ability of cancer cells to spread [52]. Also, eribulin has shown effects on ECM-related signaling pathways. Cytokine TGF-β induces fibrosis and extracellular matrix deposition; cancer cells frequently use it as a means of metastatic support. Eribulin can lessen the formation and alteration of ECM by blocking TGF-β signaling [53]. Cell–ECM interactions are mediated by cell surface receptors called integrins. By changing integrin signaling, eribulin can lessen cancer cells’ adherence, motility, and invasion of the extracellular matrix [54]. In some cases eribulin has induced changes in the expression of genes involved in ECM production and remodeling in tumor cells by reducing the synthesis of collagen and fibronectin which are major components of ECM, thereby making the ECM less supportive for cancer cells [55].

#### 3.1.4. Interaction with Signaling Pathways

**Figure 2:**
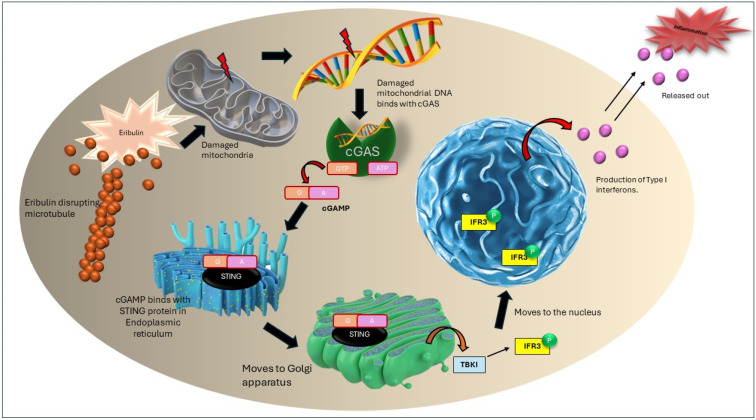
An overview of the cGAS-STING pathway initiated by microtubule destabilizer eribulin [61].

Apart from its well-known antimitotic effect, eribulin also influences various cancer-related signaling pathways. A study has investigated the impact of several microtubule-targeting drugs on triple-negative breast cancer (TNBC) cell proliferation and the PI3K/AKT/mTOR pathway. It was discovered that eribulin and vinblastine decreased p-AKT levels and inhibited cell growth. The potential cause of eribulin’s decreased phosphorylation of AKT could be the disruption of AKT localization caused by microtubule depolymerization. When eribulin and the mTOR inhibitor everolimus were combined, the survival and proliferation of cancer cells were synergistically suppressed. This suggests that eribulin and everolimus together may be able to successfully overcome resistance mechanisms and improve therapy efficaciousness in TNBC patients [56]. Another study discovered that regardless of the presence of caspase-3, eribulin and paclitaxel primarily cause caspase-independent cellular death in MCF-7 breast cancer cells. Both medications activated important apoptosis proteins, including p53, Plk1, caspase-2, Bim, and MAPKs ERK and JNK, however, these proteins were not necessary for the cytotoxic effects. Eribulin and paclitaxel exhibit similar effects on the mitotic spindle, however, they differ insignificantly in the downstream signaling of cell death in these cells [57]. Results of another study showed that in both myeloid and TNBC cells, the microtubule destabilizer eribulin, as opposed to the microtubule stabilizer paclitaxel, activates the cGAS-STING pathway (Figure 2) to stimulate the expression of interferon-β. This activation is connected to the cytoplasmic build-up of mitochondrial DNA. According to these results, eribulin may activate innate immune responses apart from its well-established impacts on cell proliferation and toxicity, offering new insight into the processes behind its actions [58].

### 3.2. Cancer-Related Therapeutic Applications and Combination Therapies

Eribulin has demonstrated noteworthy effectiveness in treating metastatic breast cancer in individuals who have previously completed numerous chemotherapy regimens. In this situation, eribulin has been approved for usage, showing better overall survival rates than alternative therapies. A study that included 513 patients has revealed that, for 78% of the total patients, eribulin was the third-line therapy, while for the remainder, it was the fourth-line therapy or later [59]. Another study revealed that eribulin can be used to avoid new metastases in metastatic breast cancer patients [60]. Many combination therapies with eribulin have been reported by several studies for breast cancers. Yamashita et al. have studied about the effects of combining eribulin with pertuzumab and trastuzumab for metastatic breast cancer and another group of researchers have studied combining eribulin with everolimus in metastatic triple-negative breast cancer [61].

Advanced liposarcoma is another kind of soft tissue sarcoma that has been approved for treatment with eribulin. For individuals with this aggressive malignancy, especially those whose disease progresses after traditional therapies, it provides a therapeutic option. By using a variety of mechanisms of action to stop tumor growth, eribulin may help patients with advanced liposarcoma achieve better outcomes when used in conjunction with other chemotherapy drugs like doxorubicin or ifosfamide [62].

The efficacy of halichondrins in treating non-small cell lung cancer is still being investigated. Early research points to possible advantages, particularly in situations where traditional treatments are ineffective. Eribulin may strengthen the immune response to non-small cell lung cancer (NSCLC), increasing response rates and survival when paired with immune checkpoint drugs like pembrolizumab. When single-agent treatments are ineffective, combining eribulin with targeted therapy such as EGFR inhibitors (erlotinib, for example) may be advantageous [63].

Early research suggests that halichondrins and their analogs can decrease tumor development and proliferation, which is why they are being studied for their therapeutic potential in prostate cancer. Combining eribulin with ADT may help treat advanced prostate cancer by focusing on several tumor biology factors. Halichondrin-based therapy may be beneficial for pancreatic cancer, a disease that is notorious for its poor prognosis and resistance to numerous treatments. The main goal of the research is to determine how these substances affect pancreatic tumor cells. Studies on halichondrins’ antitumor action are also being conducted in the context of ovarian cancer, where they may provide patients with new hope if their previous treatments have not been effective [64]. Better clinical outcomes can result from combining eribulin with platinum compounds, such as carboplatin, to increase the cytotoxic effects on ovarian cancer cells. It may be possible to reduce tumor angiogenesis and increase therapeutic effectiveness by combining eribulin with antiangiogenic drugs such as bevacizumab [65].

Overall, by utilizing synergistic effects, bypassing resistance mechanisms, and targeting various pathways involved in tumor development and progression, the combination of halichondrins such as eribulin with other therapeutic methods shows promise for enhancing the outcomes of cancer treatment.

## 4. Preclinical and Clinical Studies

The development of eribulin as a powerful therapeutic agent for cancer is supported by a carefully planned sequence of preclinical and clinical trials, which illustrates the difficult process of transferring scientific discovery into practical application. Table 2 summarizes several reported preclinical studies that were carried out on eribulin.

The purpose of eribulin clinical trials is to assess the safety and effectiveness of this artificial analog of halichondrin B. It is specifically made to block microtubule dynamics and offer therapeutic advantages to patients suffering from a variety of cancers, especially liposarcoma and metastatic breast cancer. Table 3 summarizes several clinical trials that were performed using eribulin to elaborate its potential antitumor abilities.

## 5. Challenges and Future Directions

Despite having excellent antitumor potential, halichondrin and its synthetic analogs pose several challenges in different aspects. This section will provide a glimpse of the challenges and future directions researchers must overcome and foresee.

### 5.1. Complexity of Synthesis

One of the major challenges regarding halichondrins is their synthesis due to their intricate molecular architecture. Because of their complex arrangement of rings, stereocenters, and closely spaced functional groups, these compounds necessitate exact control over stereochemistry and regioselectivity during synthesis. Large cyclic structures must be formed, which frequently requires sophisticated macrolactonization reactions, adding to the complexity [17]. Furthermore, the complete synthesis necessitates novel approaches to effectively construct and connect diverse subunits, indicating the substantial synthetic obstacles and cutting-edge methods required to accomplish these challenging molecular goals.

Due to multiple important aspects, the complete synthesis of halichondrins is a challenging task. Stereocenters, or locations in the molecule where the atoms’ spatial arrangement can change, are abundant in halichondrins. For the molecule to have biological activity, these stereocenters must be established correctly [79]. To guarantee the proper three-dimensional arrangement, this calls for extremely selective reactions and frequently the employment of chiral catalysts or auxiliaries. The complicated polyether structure of halichondrin molecules is made up of many rings that include oxygen [39]. One major synthetic problem is to construct these rings with the proper stereochemistry and in the correct order. These rings are frequently produced by complex cyclization events that call for exact control.

Several functional groups, including hydroxyls, ethers, and ketones, must be present during the synthesis and must be installed and modified without interfering with one another. To guarantee that every functional group is compatible with the synthesis’s later stages, the synthetic route must be carefully planned. Large molecule fragments are frequently built individually during the synthesis process and then joined together. To guarantee that the finished product is produced in good yield and with the right structure, this fragment coupling needs to be completed with extreme efficiency and precision. The total yield is influenced by the synthetic route’s length, which frequently consists of dozens of stages. Every stage of the synthesis needs to be optimized for yield and selectivity since, over a number of steps, even tiny inefficiencies might result in considerable losses [17].

### 5.2. Bioavailability and Pharmacokinetics

The intricate chemical architectures and biological characteristics of halichondrin and eribulin provide considerable hurdles for their pharmacokinetics and bioavailability.

Because of its massive and complicated molecular structure, eribulin may have trouble absorbing and may not be able to pass across cell membranes as easily. To guarantee sufficient bioavailability, intravenous administration is frequently required, avoiding the gastrointestinal system where absorption may be uneven and ineffective. After entering the bloodstream, eribulin needs to be transported to the intended tissues. Its distribution profile may be impacted by its substantial binding to plasma proteins due to its big size and complicated structure. Furthermore, eribulin’s capacity to infiltrate solid tumors and attain therapeutic concentrations within the malignant tissue is essential and can present a substantial challenge.

Another factor to consider is the metabolic stability of eribulin. For the medication to remain at therapeutic levels in the body, it should ideally be resistant to fast metabolic breakdown [80]. Eribulin, however, can be altered by metabolic enzymes in the liver and other tissues, which could result in harmful or inactive metabolites. For drugs to work effectively, these metabolic pathways must be understood and managed. The liver and kidneys are mainly responsible for eribulin excretion [81]. Another difficulty is making sure the medication and its metabolites are effectively eliminated without being harmful. Patients may have additional complications related to renal or hepatic impairment, necessitating dose modifications and close observation [82].

It is widely known how eribulin interacts with tubulin, its target, and how this contact alters microtubule dynamics [83]. However, elements including drug resistance mechanisms in cancer cells, the tumor microenvironment, and patient-specific characteristics like genetic variations impacting medication metabolism and response can all have an impact on how effective it is. Eribulin has a limited therapeutic window in which it can effectively combat cancer cells without producing intolerable amounts of toxicity. Achieving the ideal balance between safety and efficacy necessitates meticulous dosage calculations and close observation of the drug’s physiological levels. Eribulin may cause changed pharmacokinetics or higher toxicity if it interacts with other drugs the patient is taking. Treatment plans may become more complex because of these interactions, which call for careful assessment and management.

### 5.3. Drug Resistance

Drug resistance in the treatment of tumors when using eribulin may arise via several mechanisms. The protein that makes up microtubules, tubulin, can mutate in tumor cells. This can lower the binding affinity of eribulin, decreasing the effectiveness of the medication. Eribulin can damage DNA indirectly by interfering with the activity of microtubules, which in turn affects the mitotic spindle [84]. When a medicine damages DNA, tumor cells can become more adept at repairing it, which enables them to endure. Efflux pumps that actively move eribulin out of the cell, such as P-glycoprotein, can be upregulated by tumor cells. As a result, the drug’s cytotoxic effects are lessened by lowering its intracellular concentration [85].

Because tumor cells can alter their cell cycle checkpoints, they can evade the eribulin-induced cell cycle arrest. This permits the cells to multiply even when the medication is present. To survive the cytotoxic stress brought on by eribulin, cancer cells could activate stress response pathways such as the unfolded protein response or autophagy [86]. The efficacy of the medication may be diminished by these adaptive reactions. Drug resistance may be influenced by the tumor microenvironment, which includes stromal cells and hypoxia (low oxygen levels). Drug uptake and metabolism can be affected by hypoxia, and stromal cells can aid cancer cells to withstand the effects of eribulin by sending them survival signals [87]. Genetically diverse subpopulations of cells with varying mutations and properties frequently make up tumors. Certain subpopulations might have resistance mechanisms innately or develop them fast, which would cause resistant cells to proliferate when treated with eribulin.

### 5.4. Future Directions

The development of halichondrins and their synthetic analogs in the future will require improvements in several crucial areas to maximize their therapeutic potential and availability. Since halichondrin and its synthetic analogs have complex structures, more streamlined synthetic procedures would benefit researchers in developing cost-effective products. This can be achieved by simplifying the synthetic process, enhancing the overall efficiency and improving the yield. Optimizing the synthetic routes via green chemistry approaches would be beneficial to minimize hazardous waste and energy consumption [88].

Another potential prospect is to enhance the drug delivery system. This can be achieved through employing nanotechnological approaches including nanoparticles, liposomes, or another advanced delivery system to improve the targeting ability and bioavailability of halichondrin analogs. Synergistic combinations of halichondrin with other chemotherapeutic agents, immunotherapies, or targeted therapies to enhance efficacy and overcome resistance could be another notable prospect [89]. Discovering protein or genetic markers that indicate a person’s reaction to halichondrin analogs would allow for more individualized treatment plans. Adjusting halichondrin analog medicines based on a patient’s genetic information to maximize benefits and reduce side effects would allow study of the pharmacogenomic potentials of halichondrin and its analogs.

Conducting in-depth studies on halichondrin’s mechanism of action would be beneficial to developing new analogs and further studies on resistance mechanisms would help to find ways to overcome or prevent resistance. Implementing adaptive clinical trial designs that enable real-time adjustments depending on interim findings will speed up the creation of efficient therapies. To find novel treatment prospects, testing halichondrin analogs in cancer types beyond their existing indications is recommended [90]. Looking into the possibility of using halichondrins to treat other diseases such as infections or neurological conditions would be an interesting study focus. By concentrating on these areas, more efficient and widely available cancer treatments and possibly treatments for other illnesses can result from the development of halichondrins and their synthetic analogs in the future.

#### Biosynthetic Approaches

To synthesize complex chemicals such as halichondrin analogs, biosynthetic methods make use of the biological machinery found in nature. These methods may provide a more effective, sustainable, and selective means of producing these kinds of chemicals. One such biosynthetic strategy is gene cloning and expression. This can be implemented by isolating the biosynthetic gene clusters from the marine sponge Halichondria and cloning the genes into an easily culturable organism, such as *Escherichia coli* or *Streptomyces* species. By using this method, halichondrins can be produced in a controlled laboratory setting instead of relying on the frequently unsustainable method of natural gathering [91].

Pathway engineering is another biosynthetic strategy that involves manipulating the biosynthetic pathway in microorganisms to increase or create novel analogs of halichondrin. To do this, the native biosynthesis pathway can be altered by overexpressing important enzymes, eliminating rival pathways, or adding regulatory elements to boost yield. Alternatively, combinatorial biosynthesis can be used to introduce genes from other organisms to produce novel halichondrin analogs. In addition to facilitating the creation of novel compounds with possibly enhanced medicinal qualities, pathway engineering can optimize the production process and make it more scalable and efficient [92]. Precursor-directed biosynthesis can be achieved by supplying a microorganism or an enzyme system with tailored precursors that can be incorporated into the halichondrin framework and using enzymes with broad substrate specificity to accommodate the modified precursors. By utilizing natural biosynthetic machinery, which can be more selective and effective than chemical synthesis alone, this method enables the manufacture of specific analogs [93].

Another strategy is to engineer or find enzymes that can catalyze important processes in the production of halichondrin analogs. Implementation can be accomplished by identifying the enzymes in the pathway of halichondrin biosynthesis that oversee difficult transformations, like glycosylation, oxidation, or macrocyclization, and then engineering these enzymes to increase their activity, selectivity for new substrates, or efficiency. Enzymes are perfect for complicated synthesis because they can catalyze reactions under mild conditions and with high selectivity. Enzymes that have been engineered may open novel analogs or more effective production [94].

These complex compounds present considerable problems that can be handled by optimizing natural routes, combining them with chemical synthesis where necessary, and harnessing their potential. These techniques not only increase the productivity of the manufacturing process but also create new avenues for the development of innovative analogs with improved therapeutic potential.

## 6. Conclusions

Anticancer treatments have made tremendous progress with the development of halichondrin and its synthetic analogs. These substances, which were initially obtained from sea sponges, have shown strong cytotoxic effects on a range of cancer cell lines. The principal mode of action entails impeding the proliferation of cancer cells by disrupting the dynamics of microtubules, an essential component of cell division. To improve pharmacological qualities and get beyond the constraints of the natural product supply, synthetic analogs of halichondrin, including eribulin, have been produced. Eribulin is one example of an analog that has been licensed for the treatment of liposarcoma and metastatic breast cancer, demonstrating the promise of these drugs in medicine. In conclusion, halichondrin and its synthetic analogs are promising agents in the fight against cancer. Their unique mechanism of action and demonstrated efficacy in clinical settings underscore their potential as valuable additions to cancer treatment regimens. Ongoing research and development are likely to yield further improvements and new analogs, enhancing the therapeutic options available to patients with various forms of cancer.

## Figures and Tables

**Table 1:** General information about common halichondrin types.

	Chemical Structure	Molecular Formula	Molecular Weight (g/mol)	Sources	Synthetic Analogs	References
Halichondrin B	Consists of polyether domain and C1–C38 macrolactone domain	C_60_H_86_O_19_	1111.3	*Halichondia okadai*, *Axinells* sp., and *Phakellia carteri*	Eribulin	[22,23]
Halichondrin C	Polyether domain with an oxidation at C12. Belongs to the C series of halichondrin	C_60_H_86_O_20_	1127.3	*Halichondia okadai*	Not specified	[22,24]
Norhalichondrin B	Belongs to the B series of halichondrin	C_59_H_82_O_19_	1095.3	*Halichondia okadai* and *Lissodendory* sp.	Not specified	[9,25]
Homohalichondrin B	Belongs to the B series of halichondrin	C_61_H_86_O_19_	1123.3	*Halichondia okadai*, *Axinells* sp., and *Lissodendory* sp.	Not specified	[9,26]

**Table 2:** Preclinical studies on different cancer cells and cancer-related mouse models using eribulin.

Study ID	Study Type	Model Used	Objectives	Key Findings	References
1	In vitro	Four triple-negative breast cancer (TNBC) cell lines (MDA-MB-231, MDA-MB-468, BT-549, and MX-1)	To study the combination effect of eribulin and S-1 (5-FU)	Through eribulin’s MET induction, the combination of S-1 (5-FU) and eribulin has a synergistic antitumor effect against TNBC cell lines	[66]
2	In vitro	Two human breast cancer cell lines (MDA-MB-231 and MCF-7)	To examine the effect of eribulin on breast cancer microenvironment	Phenotypical changes in the cells after eribulin treatment. Acquired cross-resistance to other anticancer agents	[67]
3	In vitro	HeLa (human cervical cancer) cell line and FaDu (human pharyngeal carcinoma) cell line	To study eribulin’s radiosensitizing qualities in cervical and head and neck cancer cell lines and the alterations it causes to the cell cycle and apoptosis	In both cell lines, eribulin pretreatment dramatically boosted radio-induced cell death at various dosages. A range of clinically significant radiation doses cause radiosensitization, and adding the medication increases radiosensitivity more than twice as much as radiation alone	[68]
4	In vitro	Cutaneous squamous cell carcinoma (cSCC) and normal primary dermal fibroblast (NHDF)	To examine the effect of eribulin on cSCC cells and possibility of innovative therapies	In cSCC cell lines, eribulin causes irreversible mitotic blockage and death, which results in tumor regression. Suggests that eribulin may possess exceptional antineoplastic efficacy against cSCC cells, irrespective of their genetic composition	[69]
5	In vitro	Hepatocellular carcinoma (HCC) cell line	To look into how variations in microtubule acetylation levels affect treatment outcomes and HCC development	A higher amount of acetyl-α-tubulin-lys40 is associated with a decreased sensitivity to eribulin. Cells exhibiting reduced acetyl-α-tubulin-lys40 levels were more susceptible to apoptosis triggered by eribulin. The microtubule assembly was disrupted by eribulin in a dose-dependent way	[70]
6	In vivo	5–6-week-old female BALB/C^nu/nu^ mice	To study the anticancer activity of eribulin or eribulin-LF as monotherapy or in combination with anti-PD-1 Ab using a P-glycoprotein-knockout 4T1 mouse	When eribulin and eribulin-LF were administered to immunocompetent mice instead of immunodeficient mice, their anticancer activity was greater, suggesting that their immunomodulatory action was the source of their antitumor activity. When eribulin and eribulin-LF were combined with anti-PD-1 Ab, the combination therapy demonstrated anticancer effectiveness. Additionally, when eribulin-LF was used in conjunction with anti-PD-1 Ab, the administration interval and dose were larger than when eribulin was used alone	[31]
7	In vivo	4–6-week-old female^nu/nu^ nude mice	To demonstrate the sensitivity of the liver-metastasis PDOX model to low-dose o-rMETase and eribulin, as well as their combination	A significant inhibition in the TNBC growth in the liver compared to the control group after 2 weeks	[71]
8	In vivo	Mice with subcutaneous Lewis’s lung cancer (LLC) that are immunocompetent	To investigate the impact of eribulin on the cellular components of the lymphoid and myeloid lineages in the spleen and malignancies	The spleens of mice with LLC tumors that were treated with a vehicle were found to be roughly twice as large as those of mice without tumors. A strong positive correlation (r^2^ = 0.92) between the weight of the spleen and the size of each individual tumor	[72]

**Table 3:** Clinical trials that were carried out using eribulin as a whole or in combination with other drugs for cancer treatment.

Trial ID	Phase	Study Design	Patient Population	Key Findings	References
1	I	A rapid titration design incorporating real-time pharmacokinetics (PK) was used to guide dose escalation. E7389 was administered as a weekly bolus for three consecutive weeks followed by a one-week break, starting at a dose of 0.125 mg/m^2^ per week	40	The tri-phasic elimination and the prolonged terminal t1/2 of 36–48 h are shown in the pharmacokinetic data. E7389’s plasma levels are above the threshold for in vitro cytotoxicity at the MTD for more than a week. Thirteen patients were treated at the MTD, and repeated tumor samples were taken. These specimens’ fluorescent IHC analyses show that E7389 destabilizes microtubule organization in malignancies in vivo	[73]
2	I	Every 21 days, on days 1 and 8, eribulin mesylate was injected intravenously during a 5 min period. Three patient cohorts received treatment at doses of 0.7, 1.0, 1.4, and 2.0 mg/m^2^. Measurements of the tumor were taken at baseline and every six weeks. In the first cycle, pharmacokinetics were examined on days 1 through 8	15	Eribulin mesylate’s primary toxicity is neutropenia, which is easily treated. For phase II investigations, a dose of 1.4 mg/m^2^ given on days 1 and 8 every three weeks is advised	[74]
3	II	Multicenter, single-arm phase II study	28	With good safety and efficacy results, a combination regimen of trastuzumab + eribulin represents a potentially significant initial treatment option for advanced and recurrent HER2-positive breast cancer.	[75]
4	II	Open-label, multisite, single-arm ECOG trial	119	The chemo-naive stratum, the prior-taxane stratum, and the 2-prior-chemotherapy stratum had confirmed PSA response rates (50% drop from baseline) of 29% (90% [18.2%, 41.2%]; *p* = 0.20), 10% (90% [5.2%, 27.1%]; *p* = 1.00), and 4% ([0.2%, 18.3%]; *p* = 0.59), respectively. The incidence of febrile neutropenia was 4%	[76]
5	II	Single-arm, multicenter, open-label. Anthracycline- and taxane-pretreated patients received 1.4 mg/m^2^ eribulin mesylate (21-day cycle)	80	Eribulin demonstrated improved tolerability and effectiveness in patients with extensively pretreated metastatic breast cancer	[77]
6	II	Open-label, multicenter study in which the safety and efficacy of eribulin were evaluated as a first- or second-line treatment (21 day cycle)	35	In prior clinical trials, eribulin showed adequate efficacy as a first- or second-line therapy for metastatic breast cancer when compared to taxane and capecitabine treatment. Eribulin’s safety profile was deemed satisfactory	[78]

ECOG: Easton Cooperative Oncology Group.
